# Depression and Its Effect on the Menstrual Cycle

**DOI:** 10.7759/cureus.16532

**Published:** 2021-07-21

**Authors:** Jaskamal Padda, Khizer Khalid, Gazala Hitawala, Nitya Batra, Sindhu Pokhriyal, Ayushi Mohan, Ujala Zubair, Ayden Charlene Cooper, Gutteridge Jean-Charles

**Affiliations:** 1 Internal Medicine, JC Medical Center, Orlando, USA; 2 Internal Medicine, Avalon University School of Medicine, Willemstad, CUW; 3 Family Medicine, Dow University of Health Sciences, Karachi, PAK; 4 Internal Medicine, Advent Health and Orlando Health Hospital, Orlando, USA

**Keywords:** depression, menstrual cycle, menorrhagia, dysmenorrhea, antidepressants

## Abstract

A strong association is noted between depression and early perimenopause as well as menopause. The association was found to be the greatest in women with natural menopause at the age less than 40 years. Excessive corticotropin-releasing hormone (CRH) levels in depression lead to inhibition of the hypothalamic-pituitary-gonadal (HPG) axis and increased cortisol levels which further inhibits the action of gonadotropin-releasing hormone (GnRH) neurons, gonadotrophs, and gonads. The resulting changes in luteinizing hormone (LH) amplitude, follicle-stimulating hormone (FSH) levels, and LH pulse frequency were noted in patients with depression.

Besides depression, earlier surgical menopause is associated with cognitive decline. In addition, it is seen that menopausal changes predisposed females to an increased risk of depression. The association between dysmenorrhea and depression was found to be bidirectional and congruent in most studies. Patients with dysmenorrhea and coexisting depression had enhanced pain perception along with a poor response to pain relief measures. Even the treatment of underlying depression has been shown to cause menorrhagia. On the other hand, amenorrhea has also been reported as a side effect of sertraline and electroconvulsive therapy. Menstrual disorders contribute to a significant number of outpatient gynecological visits per year in the United States. Co-existing or history of depression can either be the cause of or interfere in the treatment of these disorders. Furthermore, the treatment of depression can be the etiology of various menstrual abnormalities, while menstrual disorders themselves could be the cause of depression. The increasing prevalence of depression, women’s health, multiple female-specific subtypes, and the preexisting burden of menstrual disorders necessitates more detailed studies on the effects of depression on the menstrual cycle.

## Introduction and background

Major depressive disorder (MDD) is associated with significant gender disparity. Women are afflicted with depression twice more likely than men, and it is the second leading cause of disease burden for women in the United States [1,2]. The lifetime prevalence of MDD is 26.1% among women whereas it is 14.7% among men in the United States [3]. Moreover, depressive disorders such as premenopausal dysphoric disorder and postpartum depression are gender-specific. Interaction between various factors namely neurobiological, hormonal, genetic, social, environmental has been implicated to explain these differences [4-6]. Structural differences between the brains of men and women, altered sensitivity to various neurotransmitters and hormones like serotonin, gamma-aminobutyric acid (GABA), allopregnanolone, estrogen, and corticosteroids along with genetic predisposition and cultural factors, such as lower health-seeking behaviors, social isolation, high incidence of abuse, and marital and child-rearing stressors, contribute to the development of depression in women [4-6]. This increased susceptibility is seen only during reproductive years, especially during premenstrual, pregnancy, and perimenopausal phase. The prevalence of depression in prepuberty and after the age of 55 years is almost equal between males and females [4].

Women diagnosed with MDD are more likely to experience severe disease with somatic symptoms and a higher degree of functional impairment [7-9]. One of such under-recognized yet very pertinent symptoms is menstrual cycle abnormalities, including dysmenorrhea, menorrhagia, menstrual irregularities, and premenstrual symptoms [10-12]. A study in 2012 concluded that there is an increased incidence of heavy menstrual bleeding by 1.89 times in women with a past medical history of MDD [10]. The more symptoms that a woman presents with heavy bleeding, gushing, passage of clots, the stronger the association with a history of MDD. This can be due to higher variability in hormonal levels or because of over-awareness and higher reporting of distressing symptoms [10]. Other than menstruation disorders, MDD is a risk factor for several other disorders as well, such as cardiovascular disease, diabetes, dizziness, and chronic pain. The plausible reasons for this are unhealthy lifestyles in depressed patients like smoking, alcohol, drug use, physical inactivity, sleep disturbances, non-compliance with medical regimens in addition to varied sensory perception and dysregulation of autonomic, inflammatory, and immune systems [13,14]. 

The burden of menstrual abnormalities caused by MDD should not be underestimated. Primary care physicians and gynecologists treating women with menstrual disorders should follow a multidisciplinary approach including a depression screening. Patients should be made aware of the common symptoms of depression, comorbidities, coping mechanisms, and treatment options. Close follow-up should be ensured for early detection and intervention. Moreover, the literature available on the effects of depression on the menstrual cycle is limited and most of them are over a decade old. The temporal relationship between depression and menstruation is not clearly elucidated in present studies. Given the load of depression and menstrual disorders in women, new research using the latest diagnostic criteria for depression is necessary [10]. 

## Review

Defining major depressive disorder

Major depressive disorder (MDD) is a disease that affects our mood, thought processes, and actions. Depression includes a spectrum of symptoms that categorizes the patient from mild to severe. Minimum duration of two weeks of symptoms with a decline in functioning is required for diagnosing depression. In addition, general medical conditions like thyroid disorders must be ruled out since they can present in a similar way. MDD can present with many symptoms (Table 1) [15].

**Table 1:** Symptoms of Major Depressive Disorder

Symptoms
Low mood
Anhedonia
Unintentional weight changes
Change in sleep duration
Excessive tiredness
Psychomotor retardation
Feeling blameworthy or valueless
Finding it difficult to focus and take decisions
Suicidal ideations

Depression and its effect on hormones 

**Figure 1:**
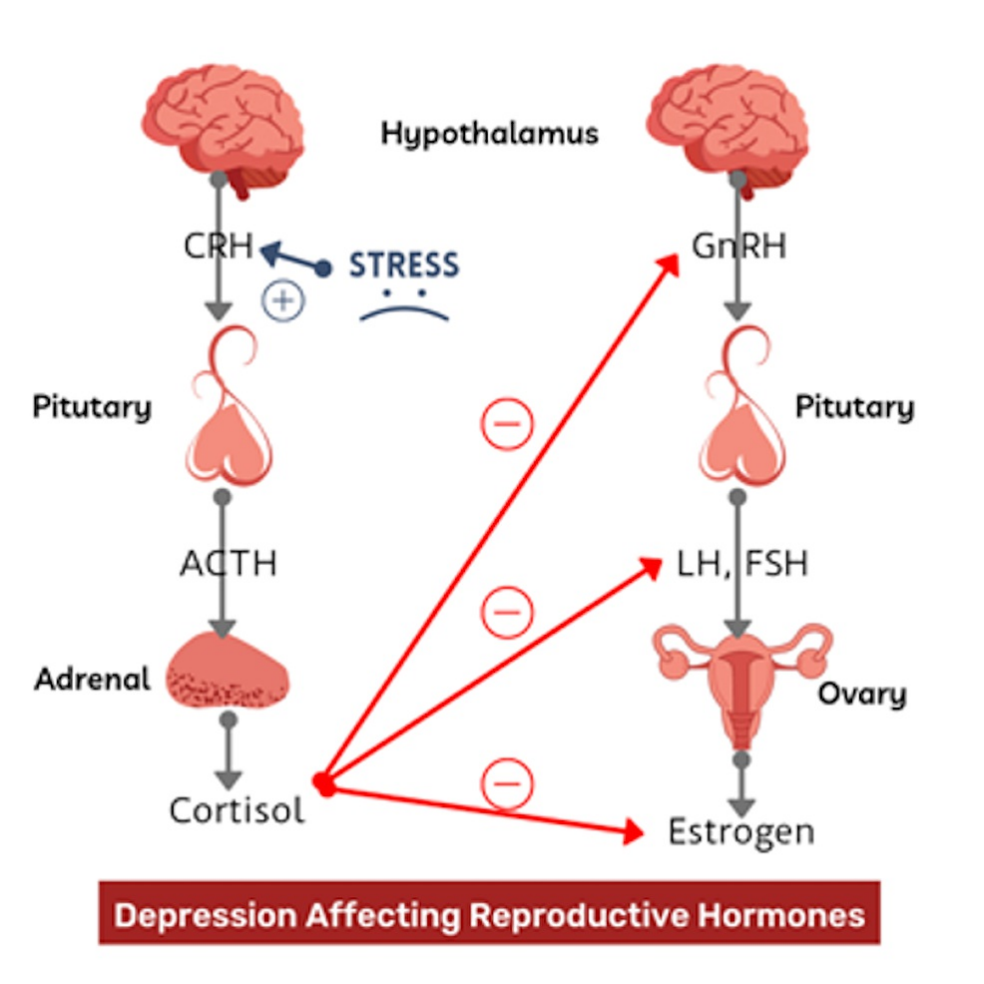
Depression Affecting Reproductive Hormones Image created by the author Gazala Hitawala.

There are multiple reports that establish the influence of reproductive hormones on depression [16-18]. Conversely, depression also plays a role in the regulation of reproductive hormones. The hypothalamic-pituitary-adrenal (HPA) axis is activated during stress, which results in the secretion of CRH from the hypothalamus. CRH acts on the pituitary, facilitating the release of adrenocorticotropic hormone (ACTH), which interacts with the adrenal cortex and stimulates the release of cortisol. In MDD, chronic stress results in a dysregulated HPA axis [19,20]. It is evident that stress-induced glucocorticoids inhibit the action of gonadotropin-releasing hormone (GnRH) neurons, gonadotrophs, and the gonads (Figure 1) [21]. Additionally, excessive CRH levels in depression lead to inhibition of the hypothalamic-pituitary-gonadal (HPG) axis [22-24].

The existing literature depicts a variable impact of depression on the gonadotropin hormones. Depression has a variant effect discreetly on the follicle-stimulating hormone (FSH) levels, mean luteinizing hormone (LH) levels, the LH pulsatility, and the estrogen levels. A study in 1997 reported that depressed women had higher LH amplitudes. However, there was no significant difference in the number of LH pulses over an eight-hour period [25]. In the year 2000, Young et al. conducted a similar study on reproductive-age women with major depressive disorder. There was no difference in the FSH levels, LH pulse frequency, and LH amplitude compared to the controls [26]. Furthermore, the reanalysis of the studies in 2003 resolved the discrepancy by confirming that LH pulsatility was decreased in the follicular phase of the menstrual cycle within depressed women of childbearing age [27]. It is evident that LH pulsatility plays a role in the regulation of the menstrual cycle. It avoids down-regulation of the GnRH receptors in the pituitary, which is crucial to maintain a normal hypothalamic-gonadal feedback loop. Furthermore, LH pulsatility determines ovulation. Thus, decreased LH pulsatility in depressed women can cause menstrual irregularities and can also affect fertility [28]. Correspondingly, a study on postmenopausal women with primary affective disorder reported decreased LH levels in the depressive phase. Moreover, decreased LH levels were also observed during the normothymic phase of the disease, suggesting that the findings were irrespective of the symptoms [29]. Another study on postmenopausal women reported 33% reduced LH concentration in postmenopausal depressed women compared to their normal counterparts. Additionally, 50% of the depressed women had LH levels lower than the lowest control value [30]. It is hypothesized that the HPG axis is disrupted in menopausal women with depression, ranging from hyperactivity in the perimenopausal period and gradually regressing to subnormal function after menopause [31].

Contrary to the effect of depression on the LH levels, the FSH levels were not significantly affected [26,32]. Studies in menopausal and perimenopausal women have reported no significant difference in the baseline FSH levels of the depressed participants [33,34]. Subsequently, in a 2003 study on women attending menopausal clinics, between the ages of 40 years and 55 years with depression, there was an observed association between the declining FSH levels and the declining Center for Epidemiologic Studies Depression (CES-D) scale scores over a six-week period. There was a proportionate association between the CES-D score and the FSH levels. In a nutshell, women with high CES-D score had high FSH levels, and vice-versa. However, with a repeated cross-sectional study, the depressed women could not be differentiated from the non-depressed women based on their FSH levels [35]. 

The mean estradiol levels are lower in the follicular phase of the menstrual cycle in women with depression [26,36]. Furthermore, a study demonstrated the effect of 17b-estradiol on the stress circuit of the brain, including the amygdala, hippocampus, and hypothalamus; it was found that during the low estradiol phase of the menstrual cycle (early follicular phase) there was robust activity in the stress circuit compared to the high estradiol phase (late follicular/ midcycle phase) in healthy individuals. However, this difference was not observed in the depressed patients, indicating that in depressed patients the estradiol capacity to evoke neuronal response was absent [37]. As stated above, it is also hypothesized that the lower estradiol levels can either be due to gonadal dysfunction or decreased LH pulse in depression [26]. 

Depression and its association with earlier menopause

It is acclaimed that menopause is marked by an array of hormonal fluctuations, decline in ovarian function, sleep disturbances, and vasomotor symptoms [38,39]. The aforementioned menopausal attributes are preceded by perimenopause. Perimenopause is the time during which the body undergoes physiological changes to progress to menopause [40]. It is pertinent that these menopausal changes in women's life are associated with mood symptoms [16,41]. As discussed ahead, it is established that depression affects the reproductive hormone regulation in females [25,26,29]. Thus, it is significant to explore the association between depression and menopause. 

The Harvard study of moods and cycles observed that women without a history of depression were at an increased risk of depressive symptoms when entering perimenopause compared to premenopausal women [42]. It was also evident in another study that females were at an increased risk of depressive symptoms and MDD when entering perimenopause [43]. Laterally, in a study following 332 depressed women and 644 non-depressed women over 36 months, it was observed that women with depression experienced early perimenopause. Moreover, there was also a correlation between the severity of depression and progression to perimenopause. Women with severe depression (Hamilton Rating Scale for Depression scores >8) had a two times faster rate of entering perimenopause compared to the non-depressed women [44].

A similar pattern was also reported of early menopause in females with a history of depression. A study reported that 14% of females with early menopause had a history of medically treated depression for at least one year, compared to 6% control (premenopausal female or naturally menopausal after 47 years of age). The association with depression was greatest in women with natural menopause at an age less than 40 years. Moreover, there were four times increased risk of early menopause with greater than three years of depression treatment [45]. A study in Canada on 13,216 women aged 45-64 years reported that women who self-reported increased depression on the Center for Epidemiologic Studies Short Depression Scale-10 (CESD-10) had experienced premature menopause with the odds ratio of 1.45 (95% CI 1.07-1.97) [46]. Parallel to the mentioned reports, in a meta-analysis, women with premature menopause (age < 40 years) had a two-fold increased risk of depression compared to their counterparts who had menopause at age ≥ 40 years [47]. In addition to it, earlier surgical menopause (hysterectomy or unilateral or bilateral oophorectomy) was also associated with cognitive decline. It was reported that women with an early age at surgical menopause had a relatively rapid decline in global cognitive function compared to older women at the time of surgical menopause. Besides, each year of earlier surgical menopause was homologous to the effect associated with six months of aging. Furthermore, a similar association was also seen with the lesser duration of the reproductive period (the period between menarche and menopause) [48]. Thus, it is explicit that there is a strong association between depression and early perimenopause together with early menopause. 

Depression and its association with menorrhagia 

Menorrhagia is a predominant complaint in women of the reproductive age group with an annual prevalence rate of 53 per 1000 women, contributing to 20-30% outpatient gynecological visits per year in the United States [49]. About 45% of hysterectomies are performed for the treatment of menorrhagia [50]. It is a consensus that menorrhagia is caused by gynecological pathology, but it can also be a presentation of depression. Though menorrhagia and other forms of gynecological morbidities can precipitate depression, there are few studies indicating that history of depression may precede heavy menstrual bleeding [10,12,51-53]. It has been well established that stress can lead to menstrual cycle changes by altering HPG and HPA axis [54,55].

In 2012, a study conducted on women aged 42-52 years demonstrated that 67.7% of women reported heavy bleeding. The rate of past and recent major depression was significantly higher among these women, 39% and 14%, whereas it was lower for women reporting no menorrhagia, 24% and 8%. A dose-effect relation was also established, with a stronger association between past depression and a number of menorrhagia symptoms reported (heavy bleeding, flooding/gushing, and passage of clots) [10]. Another study done on 126 North Korean women in South Korea showed that the prevalence of depression was 40%, and of that, menorrhagia was 19.8%. It also revealed that depression and the number of menstrual complaints were significantly correlated. This was explained by higher levels of blood bradykinin and prostaglandin associated with stress, which causes dilatation and increase in flow in pelvic blood vessels and hence causing menorrhagia and pelvic pain [12]. One study which analyzed the data from the 2002 national Health Interview Survey determined that 19% of women experienced menstrual problems including menorrhagia, dysmenorrhea, and premenstrual syndrome and they are twice more likely to suffer from depression and anxiety than those without menstrual complaints. Additionally, they are 1.7 to three times more likely to report sadness, hopelessness, insomnia, and nervousness [51]. Segregation into cases vs non-cases was based on a 60-item general health questionnaire [53]. 

Depression can not only precede but can also hinder the treatment for menorrhagia. A study from 2007 reported that mild depression, six months from baseline, was associated with discontinuation of levonorgestrel-releasing intrauterine system treatment, effective treatment of menorrhagia, and led to higher hysterectomy rates in this group [56]. Even the treatment of underlying depression can cause menorrhagia. A case report from 2010 described an adolescent girl with MDD who developed menorrhagia three weeks after treatment with sertraline [57]. Another case report from 1992 illustrated a 40-year-old woman who developed heavy menstrual bleeding one month after commencing fluoxetine and spontaneous ecchymoses in two months [58]. Her symptoms subsided four days after stopping the medication. Amenorrhea has also been reported as a side effect of sertraline and electroconvulsive therapy [59,60].

Depression and its association with dysmenorrhea

Dysmenorrhea, which is the most common menstrual disorder seen in reproductive women, can be primary or secondary based on its etiology. Primary dysmenorrhea is classified as painful menstrual cramps without any pelvic pathology and is the more common variant, while secondary dysmenorrhea is associated with pelvic pathologies such as endometriosis, fibroid uterus, etc. [61,62]. There is considerable overlap in the definitions of dysmenorrhea and premenstrual syndrome (PMS). PMS-like syndrome refers to the presence of at least one mood symptom (anxiety, mood lability, irritability, marked anger, dysthymia) and/or physical symptom (abdominal cramps, back pain, joint pains, mastalgia, headaches, weight gain, feeling bloated) [63]. Three percent to 33% of women suffering from dysmenorrhea and up to 8% of women with PMS face issues with normal functionality of their everyday life [64,65]. The present systematic review has been designed to focus on the association between dysmenorrhea and depression. 

The association between dysmenorrhea and depression was found to be bidirectional and congruent in most studies. Rodrigues et al. conducted a study of 274 adolescents and young adults with menstrual abnormalities. It mentioned depression as the most common limitation affecting the quality of life of these young women (42.5%), whereas other studies described how the simultaneous presence of depression and dysmenorrhea pointed towards enhanced pain perception along with poor response to pain relief measures in this patient population [66].

Pain and depression have had a well-known association. American psychologist, Mark Jensen, has discussed the strong association of depression and idiopathic pain disorders and described the successful use of cognitive as well as behavior therapies in treating pain [67]. Jarvik et al. in their study reported depression to be the strongest predictor of incident back pain compared to any other clinical or anatomical predictors [68]. 

Not surprisingly, painful menses or dysmenorrhea also seems to have a convincing association with depression. Lacovides et al. in his critical review in 2015 proposed an alternative hypothesis for increased pain sensitivity in severe dysmenorrhea. They suggested that an increased level of prostaglandins triggering uterine muscle contractions alone cannot explain the pain in dysmenorrhea. They went on to say that increased central sensitivity to pain is probably what leads to enhanced peripheral response to pain in patients with severe dysmenorrhea [69]. Balik et al. in their study on 159 adolescent girls found the prevalence of dysmenorrhea to be 67.9% and described the Beck Depression score (BDS) in these patients to be averaging at 19.1+/-11.85 [70]. A Turkish study conducted by Unsal et al. also reported a positive correlation between the severity of dysmenorrhea and mean BDS (p < 0.05). They also reported a higher prevalence of depression in females experiencing dysmenorrhea compared to those who didn’t have dysmenorrhea [71].

In another relevant study on women attending the gynecological clinic for menstrual problems such as PMS, menorrhagia, and dysmenorrhea, there were two important conclusions drawn. The first was in sync with several previous studies and stated that severity of depression was directly proportional to the severity of dysmenorrhea. Second, they found a link between history of treated depression and the severity of menstrual problems [72,73].

These studies are in line with those by Pedron-Neuvo et al. and many others in which depression has been found to have a strong association with menstrual pain. However, more studies are needed to establish the mechanism by which depression causes dysmenorrhea [74].

In addition, interpreting pain objectively is a challenge in itself, as a woman's observation of pain may differ based on their personalities, social circumstances, life experiences, and several other occult influences. Although sophisticated imaging techniques such as functional magnetic resonance imaging (fMRI) have made it possible to map regions in the brain associated with painful conditions such as dysmenorrhea, these areas need more studies to help us get a better understanding of the subject [75].

Depression and its association with premenstrual syndrome

Right before women get their periods, they may experience a range of symptoms which are classified as premenstrual syndrome (PMS) or premenstrual dysphoric disorder (PMDD) depending on whether they are mild or severe. PMS includes symptoms like bloating, mood swings, feeling sad or hopeless, inability to concentrate, breast soreness, fatigue, and headaches. When these symptoms become so severe that they interfere with day-to-day functioning, they are called PMDD [76]. Various studies have cited the incidence of clinically significant PMS to be up to 8% and PMDD to be around 2% [77,78].

Several studies on neuropsychobiology like that by Taylor et al. and Rapkin et al. have shown that patients with PMS and PMDD have lower circulating levels of serotonin [79,80]. This has been hypothesized to be secondary to the cyclic hormone changes that occur during the luteal phase of menstrual cycle owing to the altered neurotransmitter production following these cyclical hormone changes [81].

It is well-known that depression is also associated with decreased circulating serotonin levels and the use of selective serotonin reuptake inhibitors (SSRI) in the treatment of depression has been an established way of treating both depression and PMDD [82]. The American Psychiatric Association has included PMDD as one of the subtypes of depression [83]. The notable clinical feature that separates the two clinical entities is the typical association of the depressive symptoms of PMDD with the luteal phase of the menstrual cycle [76].

Effect of antidepressants and ECT on menstrual cycle

Antidepressant medications are a broad and expansile group of medications that include SSRIs, serotonin-norepinephrine reuptake inhibitors (SNRIs), tricyclic antidepressants (TCAs), and monoamine oxidase (MAO) inhibitors. Several herbal supplements like St. John’s Wort are also commonly used over the counter to combat depressive symptoms. The relationship between menstrual abnormalities and antidepressants is a relatively less common phenomenon and continues to surprise physicians in daily practice [58].

A multicentric, cross-sectional study conducted by Uguz et al. analyzed 793 women on antidepressants and 639 women not on any medications. They reported that menstrual disorders were more common in women taking antidepressants with an incidence of 14.5% and the most implicated antidepressants belonged to the SSRI and SNRI subgroups. Mirtazapine was also implicated especially when it was used in combination with SSRIs and SNRIs [84].

Paradoxically, while SSRIs are the first-line drugs of choice used by many practitioners for the treatment of PMS-like symptoms, they can also rarely cause menstrual irregularities like amenorrhea. Albeit rare, the endocrine and reproductive adverse effects of SSRIs have been reported in several studies [85,86].

Kim and Park conducted a cross-sectional pilot study, in which they measured prolactin levels in all patients who had received SSRI monotherapy for a mean of 14.75 months. They concluded that although SSRI therapy can induce hyperprolactinemia, there was no correlation between the dosage of SSRIs nor did the prevalence of hyperprolactinemia vary amidst different SSRIs [87]. Although very rare, there have been case reports of SSRIs affecting thyroid function which are well-known to be associated with menstrual irregularities [88]. Similar to SSRIs, duloxetine, which is an SNRI commonly used to treat depression when it occurs in combination with conditions like diabetic neuropathy and fibromyalgia has also been associated with hyperprolactinemia and amenorrhea [89]. The mechanisms suggested in SSRI- and SNRI-induced hyperprolactinemia are by direct inhibition of the tuberoinfundibular pathway, as well as by possible direct stimulation of postsynaptic serotonergic receptors [58].

Another frequently used herb for treating depression is St John’s Wort (SJW) which is a well-known inducer of cytochrome P450 3A4 9(CYP3A4) and hence known for its interactions with several drugs including oral contraceptive pills (OCPs) and warfarin. SJW, by increasing the activity of CYP3A4, reduces the plasma levels of estrogen causing withdrawal bleeding of the endometrium. Breakthrough menstrual bleeding is therefore a frequently encountered side effect of this herb when used alongside OCPs [90]. SJW also interacts with SSRIs in the very same way as many serotonergic drugs like tramadol. It is known to increase the serotonin levels in the brain and can cause moderate serotonin syndrome in patients taking both SSRIs and SJW concurrently [91].

Finally, another frequently forgotten adverse effect is that of electroconvulsive therapy (ECT) causing amenorrhea. ECT is a safe and effective modality for managing mental health conditions like major depression and bipolar disorder which have not responded to medications and psychotherapy. ECT is rarely used to treat these refractory mental health conditions but remains a viable option in mental health treatment. Michael, who studied 687 female in-patients undergoing ECT, reported that the most frequent pattern seen during ECT was amenorrhea followed by a shortening of the first cycle, with a following longer cycle of more than 30 days. He also reported a remarkable increase in the length of the menstrual cycle in women who received between 10-20 shock treatments [92,93]. Thankfully these adverse effects are transient and benign. The exact mechanism of this is still unknown but several studies have pointed towards robust but transient hyperprolactinemia that occurs during therapy [92,93].

## Conclusions

In this study, we can conclude that depression and the menstrual cycle are interconnected, and abnormalities in either of them can affect the other. Hormonal changes in the body during menopause predispose them to depression. Depression alters the hypothalamic-pituitary axis which leads to menstrual irregularities. Our results demonstrate that women with depressive disorders have a faster rate of progression to menopause. Menorrhagia and depression are also linked to each other, with depressed patients experiencing more severe symptoms. Depression increases sensitivity to pain-causing dysmenorrhea and thus hinders the management of menorrhagia and dysmenorrhea. Further studies should be conducted to identify the mechanism by which depression causes dysmenorrhea.

Women undergoing disturbances in the menstrual cycle should be screened for depression and similarly, women experiencing depressive disorders should be evaluated for irregularities in menstruation. Antidepressants have not been proven to be helpful in these patients. Stabilizing the menstrual cycle and providing psychological support using behavioral therapy and coping strategies can help improve the quality of life of these patients. Further research is required in this modality to explain the pathways interconnecting depression and the menstrual cycle in order to come up with treatment strategies in helping women.

MDD-induced menstrual abnormalities should not be overlooked. A multidisciplinary approach, including screening to rule out MDD, should be applied by the primary care physician and gynecologist when treating women with menstrual disorders. Such patients should also be educated on common symptoms of depression, comorbidities, coping mechanisms, and treatment options. Close follow-up should be provided for immediate diagnosis and treatment. Treatment for underlying depression has been affiliated with menorrhagia, while amenorrhea has been reported with sertraline and electroconvulsive therapy. Further investigations should also be conducted on discovering an effective way to treat depression in women without causing menstrual abnormalities.
